# A clinical validation of the MR‐compatible Delta^4^ QA system in a 0.35 tesla MR linear accelerator

**DOI:** 10.1002/acm2.13216

**Published:** 2021-03-05

**Authors:** Vimal Desai, John Bayouth, Jennifer Smilowitz, Poonam Yadav

**Affiliations:** ^1^ Department of Human Oncology School of Medicine and Public Health University of Wisconsin‐Madison Madison WI USA

**Keywords:** diode‐arrays, IMRT QA, MR‐Lina, plan complexity metrics

## Abstract

**Purpose:**

To validate an MR‐compatible version of the ScandiDos Delta^4^ Phantom+ on a 0.35T MR guided linear accelerator (MR‐Linac) system and to determine the effect of plan complexity on the measurement results.

**Methods/Materials:**

36 clinical treatment plans originally delivered on a 0.35T MR linac system were re‐planned on the Delta^4^ Phantom+ MR geometry following our clinical quality assurance (QA) protocol. The QA plans were then measured using the Delta^4^ Phantom+ MR and the global gamma pass rates were compared to previous results measured using a Sun Nuclear ArcCHECK‐MR. Both 3%/3mm and 2%/2mm global gamma pass rates with a 20% dose threshold were recorded and compared. Plan complexity was quantified for each clinical plan investigated using 24 different plan metrics and each metric’s correlation with the overall 2%/2mm global gamma pass rate was investigated using Pearson correlation coefficients.

**Results:**

Both systems demonstrated comparable levels of gamma pass rates at both the 3%/3mm and 2%/2mm level for all plan complexity metrics. Nine plan metrics including area, number of active MLCs, perimeter, edge metric, leaf segment variability, complete irradiation area outline, irregularity, leaf travel index, and unique opening index were moderately (|r| > 0.5) correlated with the Delta^4^ 2%/2mm global gamma pass rates whereas those same metrics had weak correlation with the ArcCHECK‐MR pass rates. Only the perimeter to area ratio and small aperture score (20 mm) metrics showed moderate correlation with the ArcCHECK‐MR gamma pass rates.

**Conclusions:**

The MR‐compatible version of the ScandiDos Delta^4^ Phantom+ MR has been validated for clinical use on a 0.35T MR‐Linac with results being comparable to an ArcCHECK‐MR system in use clinically for almost five years. Most plan complexity metrics did not correlate with lower 2%/2mm gamma pass rates using the ArcCHECK‐MR but several metrics were found to be moderately correlated with lower 2%/2mm global gamma pass rates for the Delta^4^ Phantom+ MR.

## 1 Introduction

Intensity modulated radiation therapy (IMRT) is a highly conformal external beam cancer treatment technique. Various factors such as commissioning data, dose calculation algorithm, delivery process, and performance of treatment delivery components have contributed to uncertainties in radiation therapy treatment. Patient‐specific quality assurance (QA) for IMRT plans is often mandatory^1^ and can be time consuming and laborious. As integrated linear accelerator and magnetic resonance imaging (MRI) hybrid system like Unity from Elekta (Elekta, Stockholm, Sweden) and MRIdian® from ViewRay® (ViewRay Inc, Cleveland, OH) continue to increase in popularity, QA solutions which are both MR‐compatible and appropriate for IMRT also become necessary. The use of IMRT QA tools such as point detectors, arrays, film, etc. are well documented in the literature.^2^ These same tools, however, which were previously incompatible with MRI are now being updated for use with MR‐linacs.

When considering IMRT QA solutions the desirable properties of measurement devices include high resolution, the ability to calculate dose distributions in three dimensions, easy setup, wide clinical applicability (i.e., not tailored to only one delivery modality), negligible angular and energy dependence, a large active region, and the ability to be calibrated for and measure absolute dose. Although there is much data regarding the performance of IMRT QA detectors in conventional linear accelerators (linacs), the same cannot be said of MR‐compatible versions of many of those same detectors. The presence of a magnetic field can impact the measurements from IMRT QA devices designed for use with conventional linear accelerators,^3^, ^4^ so MR‐compatible radiation detectors are not as ubiquitous as conventional radiation detectors. In addition to ionization chamber specific studies, other groups have investigated the use of radiographic film,^4^ cylindrical diode arrays^4^, ^5^ (i.e., ArcCHECK‐MR), planar diode arrays,^6^, ^7^ GAFChromic™ EBT3 film,^8^ gel dosimetry^9^ for IMRT QA on MR linac systems. The ScandiDos Delta^4^, has been commercially available since 2015 and existing literature describe its characterization and commissioning^10^ but an MR‐compatible version of the detector was only recently made available in 2019. Therefore, relatively little data exists with regards to the performance of this newer MR compatible model.

A prototype MR‐compatible Delta^4^ Phantom+ was previously characterized by de Vries et al.^11^ with regards to basic detector characteristics such as measurement reproducibility, dose linearity, field size dependence, dose rate dependency, and angular dependence. These quantities were compared to a conventional Delta^4^ Phantom+ and both devices were found to have consistent and clinically acceptable characteristics. No clinical data, however, was used in that previous work to assess the prototype Delta^4^ Phantom+ MR performance. Recently, investigators at the Miami Cancer Institute (MCI) released a white paper detailing several aspects of the Delta^4^ Phantom+ MR equipment, commissioning process, and measurement results for 14 clinical cases also treated on a ViewRay MRIdian linac.^12^ This current work expands upon the MCI white paper by evaluating the performance of the Delta^4^ Phantom+ MR using 36 clinical plans spanning a broad range of fluence modulation, target sizes, and anatomical sites of treatment on a ViewRay MRIdian 0.35T MR‐linac system. The results from the Delta^4^ Phantom+ MR are directly compared to measurements of the same plans previously conducted using an MR‐compatible Sun Nuclear ArcCHECK® (Sun Nuclear Corporation, Melbourne, FL) phantom. Finally, a series of fluence descriptive modulation complexity metrics were also calculated for each plan investigated in this work to determine which metrics, if any, might impact IMRT QA failure rates for both devices.

## 2 Materials and Methods

The MRIdian Linac system from ViewRay consists of a 6 MV flattening filter free linear accelerator sandwiched between 0.35 Tesla split superconducting magnet with a 28 cm gap between the two magnets.^13^ Its MLCs are double stacked at the isocenter plane. The minimum and maximum programmable field sizes are 0.2 × 0.415 cm^2^ and 27.4 × 24.1 cm^2^ respectively. The MRIdian system uses a step‐and‐shoot intensity modulated radiation therapy technique to deliver dose that is calculated with a Monte Carlo algorithm.^13^

The Sun Nuclear ArcCHECK‐MR phantom is comprised of 1,386 diode detectors arranged in a helical pattern and spaced 10 mm apart around a cylindrical water‐equivalent body. The length and diameter of the phantom are both 21 cm. The phantom also contains a cavity 15 cm in diameter which can accept various tissue equivalent inserts.

The ArcCHECK‐MR used in this work included a relative calibration performed by the manufacturer and the absolute calibration was performed at our institution using a NIST‐traceable A1SL scanning chamber (Standard Imaging, Middleton, WI) in a water tank at a calibration depth representative of the ArcCHECK‐MR geometry. A 9.96 cm × 9.96 cm field size was used to irradiate the chamber at a depth of 3.3 cm (to match inherent ArcCHECK‐MR buildup) in water at a source to surface (SSD) distance of 86.3 cm. 200 MU were delivered to the ionization chamber in this configuration and the chamber response was fully corrected for the effects of temperature, pressure, electrometer response, recombination, polarity, beam quality, and beam output in order to establish a known dose under reference conditions. The ArcCHECK‐MR phantom was connected via ethernet to a Windows 8 workstation running the SNC Patient software version 8.2.0.1815.

The Delta^4^ Phantom+ MR is comprised of two planar circuit boards arranged in an orthogonal crossed array pattern. The boards contain 1069 disc‐shaped, p‐type, Si diodes as the radiation detecting elements which are spaced 5 mm apart in the central high‐resolution and are spaced 10 mm apart elsewhere. The central 60 mm x 60 mm of the boards comprise the high‐resolution region while the total detector plane area is 200 mm x 200 mm. The phantom is cylindrically shaped with a diameter of 220 mm and a length of 400 mm. It is made of PMMA with a mass density of 1.19 g/cm^3^. The stated dose resolution of the Delta^4^ Phantom+ MR is 0.1 mGy with a minimum detectable dose of 1 mGy and no maximum dose limit.

An absolute dose calibration for the Delta^4^ Phantom+ MR was performed on a ViewRay MRIdian® 0.35T MR‐Linac system using the procedure recommended by the manufacturer. The same calibrated Exradin A1SL scanning chamber used to calibrate the ArcCHECK‐MR was placed into the Delta^4^ Phantom+ MR using a custom insert and used to calibrate the phantom diodes. A total of 100 monitor units (MU) were delivered to the chamber using both 9.96 × 9.96 cm^2^ and 19.92 × 19.92 cm^2^ field sizes from gantry angles of 0° and 90° for each energy investigated. All chamber charge readings were fully corrected for the effects of temperature, pressure, electrometer response, recombination, polarity, beam quality, and beam output to determine the absorbed dose. These absorbed dose readings were then entered into the Delta^4^ software to establish an energy‐ and detector‐specific calibration.

Communication with the Delta^4^ Phantom+ MR was facilitated by a wirelessly connected router inside the treatment room which itself was hardwired via ethernet to a workstation in an adjacent control room. The ScandiDos Delta^4^ software (November 2019 1.00.0180) was used which improved compatibility with the step‐and‐shoot delivery of the MR‐linac (i.e., it precluded the need to stop and start measurements between individual beam segments). Table 1 compares select properties of both the Delta^4^ Phantom+ MR and the ArcCHECK‐MR.

**Table 1:** Comparison of the Delta^4^ Phantom+ MR system to the ArcCHECK‐MR system.

Detector	Delta^4^ Phantom+ MR	ArcCHECK‐MR
Manufacturer	ScandiDos	Sun Nuclear
Diode Type	P‐type silicon diodes	N‐type silicon diodes
Number of Diodes	1069	1386
Diode Arrangement	Crossed Array	Helical
Detector Spacing	0.5 cm (high‐res) and 1 cm	1 cm
Array Diameter	20 cm	21 cm
Array Length	20 cm	21 cm
Phantom Material	PMMA	PMMA
Detector Stability*	<0.1% / kGy	0.5% / kGy

* Reported at 6 MV for both detectors.

A total of 36 clinical plans were used to assess the performance of the device. These plans spanned several anatomical treatment sites previously treated on the ViewRay MRIdian linac including abdomen (13), lung (7), liver (9), and kidney (7). The abdomen group was comprised of abdominal sarcoma plans as well as pancreas treatments. At the University of Wisconsin, the ArcCHECK‐MR phantom has historically been used for patient‐specific IMRT QA of all clinical plans. Among the QA plans selected for this work were several with global gamma pass rates of less than 97% at 3%/3mm with a 20% dose threshold when previously measured on our ArcCHECK‐MR system. Those plans were of special interest since most clinical plans on the ArcCHECK‐MR pass the 3%/3mm global gamma test at levels of 99% or higher. All QA plans were generated by transferring clinical plans to the ArcCHECK‐MR geometry and recalculating them in the ViewRay treatment planning system (TPS). In the TPS, the magnetic field was set to ON, a grid resolution of 0.2 cm was used, and uncertainty during dose predication was set to 0.5%. Delta^4^ Phantom+ MR QA plans in this work were generated in an identical manner, and in both cases, the geometric center of each phantom was placed into regions of high dose and low gradient to ensure that the high dose target region was being sufficiently sampled by the physical measurement.

Each QA plan was then delivered to the Delta^4^ Phantom+ MR system. Dose differences at levels of 2% and 3% along with distance to agreement (DTA) values of 2 mm and 3 mm were recorded as well as 2%/2mm and 3%/3mm global gamma pass rates using a 20% dose threshold. These results were then compared to previous patient‐specific QA measurements conducted at our clinic using the ArcCHECK‐MR system.

For each treatment plan investigated, 24 modulation complexity metrics were calculated to determine whether any specific metric or metrics correlated with the gamma pass rates. The metrics and means of calculation are summarized in Table 2 based on the work of Desai et al.^14^ The small aperture score (2mm) metric was not calculated in this work since no clinical plans investigated involved MLC opening widths of 2 mm or smaller. An in‐house Matlab® (Mathworks, Natick, MA) script was used to record and calculate all metrics by extracting data from the headers of the DICOM‐RT plan files.

**Table 2:** Summary of complexity metrics calculated modified from the work of Desai et al.^14^ with permission from Medical Physics.

Metric	Description
Aperture Area	Cumulative area
Number of Active Pairs	Total number of active MLC pairs engaged
Aperture Perimeter	Cumulative perimeter
Edge Metric^16^	Cumulative perimeter ignoring contributions from leaf tips
Average Leaf Pair Opening^17^	Average leaf pair opening
Complete Irradiation Area Outline	Aperture area based on the max open position of all MLCs across all control points
Aperture Area Variability^18^	MU‐weighted ratio of each control point aperture area to the CIAO
Leaf Segment Variability^18^	Variation between MLC neighbors in the same leaf bank normalized to largest difference encountered in the bank
Aperture Irregularity^15^	Noncircularity of an aperture
Unique Opening Index	Number of noncontiguous MLC‐defined openings
Cross‐Axis Score^19^	Proportion of MLCs which cross over midline
Small Aperture Score (5 mm)	Proportion of MLC pairs with separation of less than 5 mm
Small Aperture Score (10 mm)	Proportion of MLC pairs with separation of less than 10 mm
Small Aperture Score (20 mm)	Proportion of MLC pairs with separation of less than 20 mm
Mean Aperture Displacement^20^	Displacement of the aperture opening from midline
Perimeter to Area Ratio	Ratio of perimeter to area
Modulation Complexity Score^18^	Product of LSV and AAV
Leaf Travel Index^21^	Total leaf travel normalized to 10 cm
Leaf Travel Index Modulation Complexity Score^21^	Product of LTI and MCS
Closed Leaf Score	Proportion of closed MLCs within jaw‐defined field
Number of Control Points	Total number of control point segments per beam
MU per segment	Fraction of beam MU delivered per control point
Total MU	Total MU delivered per beam

The ViewRay MRIdian makes use of a stacked MLC configuration, containing an upper bank with 70 MLC pairs and a lower bank containing 68 MLC pairs. The MLC widths project to 0.83 cm at isocenter but the two sets of banks are staggered such that pseudo high resolution leaves can be generated with widths of 0.415 cm projected to isocenter. Although complexity metrics could be calculated independently for each set of MLC banks, the aperture‐specific complexity metrics in this work were calculated as if each aperture was created by a single MLC unit containing 70 pairs of MLCs with 0.415 cm widths projected to isocentre.

**Fig. 1:**
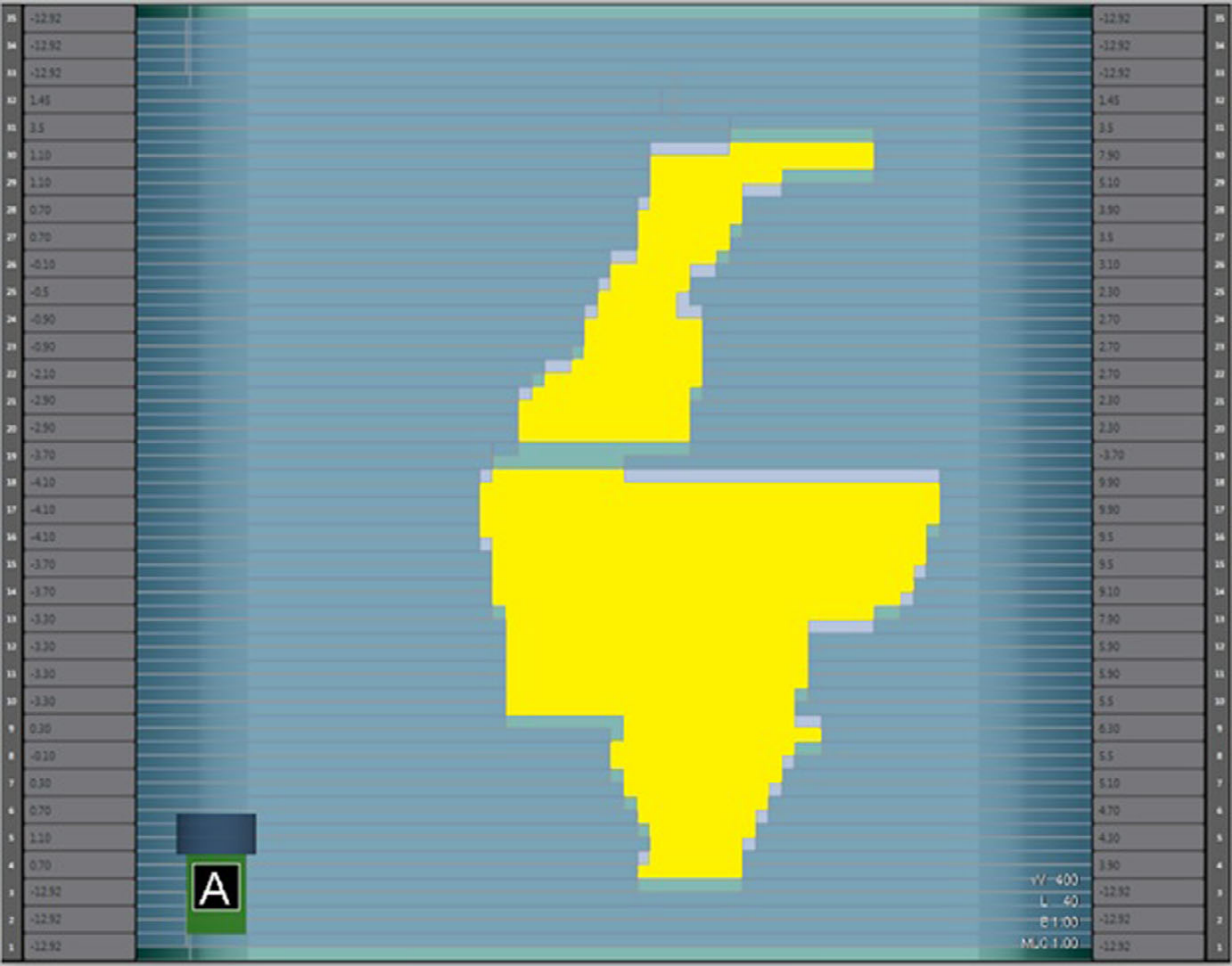
An example aperture generated by the ViewRay stacked MLC banks staggered by one half leaf width. Rather than calculating complexity metrics separately for each individual leaf bank, the composite shapes (highlighted in yellow) were instead analyzed as if they were created by 70 independent pairs of MLCs because the shapes of the MLC‐defined fluences are assumed to be more relevant to complexity metric calculations.

Importantly, this consideration affects the calculation of a few complexity metrics such as the leaf travel index and leaf segment variability which are more tailored to MLC designs containing only a single set of MLC banks (i.e., the motion of the physical MLCs on the MRIdian linac do not necessarily match the motion of the pseudo 0.415 cm MLCs which affect complexity metric calculations). Because the metrics investigated are primarily concerned with the shape of each segment’s fluence, this treatment of ViewRay MLCs was thought to be more appropriate than calculating complexity metrics for each physical set of MLCs separately. Figure 1 illustrates an example ViewRay aperture and how the resulting composite shape, as opposed to individual leaf banks, are analyzed in this work.

The 2%/2mm gamma pass rates for both devices were then investigated as a function of the various complexity metrics to see which metrics, if any, correlated with the overall pass rate. This process was not repeated for the 3%/3mm gamma pass rates because the variability of the 3%/3mm results was minimal for both devices, with almost all plans passing well above our institution’s criterion of a 95% global gamma pass rate at 3%/3mm with a 20% dose threshold. In each instance, a Pearson correlation coefficient was determined for the 2%/2mm gamma pass rate as a function of the specified complexity metric.

## 3 RESULTS

**Fig. 2:**
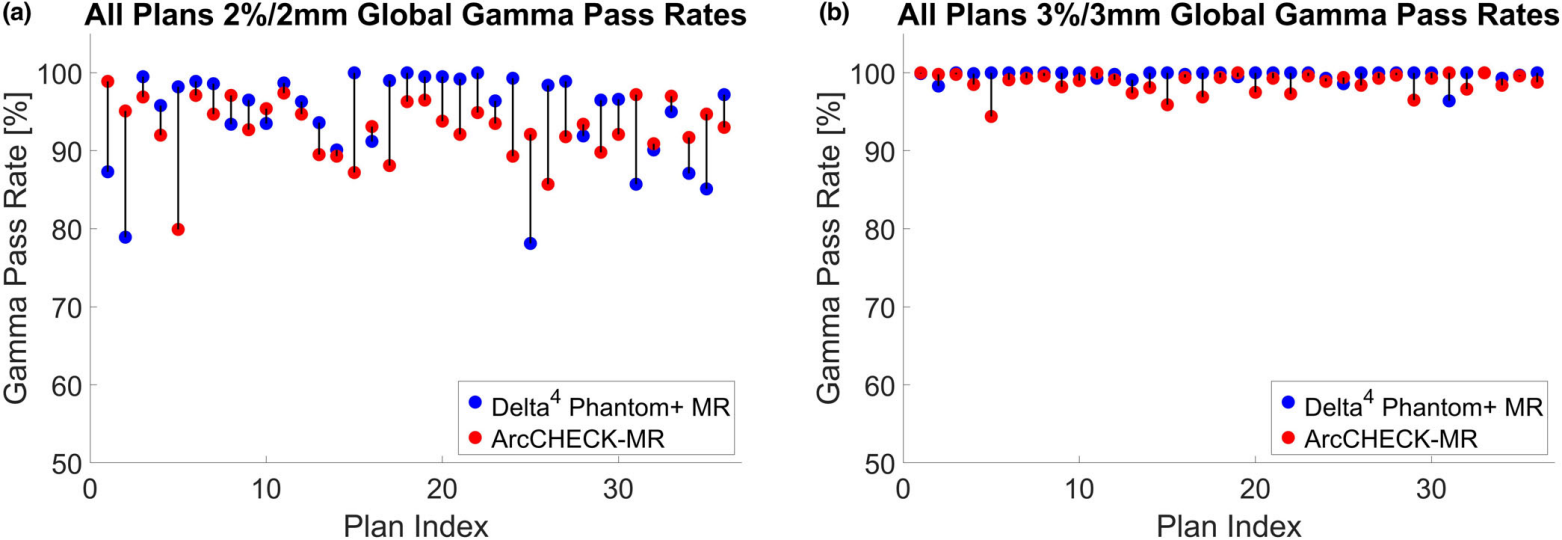
Comparison of (a) global gamma 3%/3mm pass rates and (b) global gamma 2%/2mm pass rates across all investigated plans for both the ArcCHECK‐MR as well as the Delta^4^ Phantom+ MR.

Figure 2 compares the global gamma pass rates for every plan investigated for both the Delta^4^ Phantom+ MR as well as the ArcCHECK‐MR. Tables 3 and 4 summarize the 2%/2mm and 3%/3mm gamma pass rate statistics respectively.

**Table 3:** Relevant statistics of 2%/2mm global gamma pass rates between the Delta^4^ Phantom+ MR and ArcCHECK‐MR.

2%/2mm Global Gamma Pass Rate						
Site	Delta^4^ Phantom+ MR			ArcCHECK‐MR		
	Mean	Std	Min	Mean	Std	Min
Abdomen	91.0	6.1	78.9	92.8	5.0	79.9
Lung	94.2	7.5	78.1	88.5	2.7	83.7
Liver	98.3	2.9	91.2	92.8	3.3	87.2
Kidney	96.6	2.4	93.4	95.6	1.7	92.7

**Table 4:** Relevant statistics of 3%/3mm global gamma pass rates between the Delta^4^ Phantom+ MR and ArcCHECK.

3%/3mm Global Gamma Pass Rate						
Site	Delta^4^ Phantom+ MR			ArcCHECK‐MR		
	Mean	Std	Min	Mean	Std	Min
Abdomen	99.4	1.0	96.4	98.7	1.6	94.4
Lung	99.7	0.6	98.6	97.4	0.9	96.2
Liver	99.9	0.2	99.5	98.4	1.5	95.9
Kidney	99.9	0.3	99.3	99.2	0.6	98.2

Table 5 summarizes each complexity metric’s correlation with the 2%/2mm global gamma pass rates for both the Delta^4^ Phantom+ MR as well as the ArcCHECK‐MR. In Table 5, correlations are calculated across all 36 clinical plans making no distinction between anatomical sites of treatment. Only absolute correlation values of at least 0.5 (moderately correlated) were considered relevant. In this work, any *p*‐value less than 0.05 was assumed to be statistically significant. All metrics which were at least moderately correlated with the gamma pass rates and involved an associated *p*‐value of less than 0.05 are highlighted in bold in Table 5. Table 5 demonstrates that the 36 clinical plan 2%/2mm global gamma pass rates only showed significant negative correlation with 2 of a possible 24 metrics for the ArcCHECK‐MR whereas the Delta^4^ Phantom+ MR showed significant negative correlation with 8 of 24 metrics and significant positive correlation with an additional 1 of 24 metrics.

**Table 5:** Summary of Pearson correlation coefficients with the 2%/2mm global gamma pass rate for both the ArcCHECK‐MR and the Delta^4^ Phantom+ MR. Correlation values with a magnitude larger than 0.5 and an associated p‐value of < 0.05 are highlighted in bold.

Metric	All Plans			
	ArcCHECK‐MR		Delta^4^ Phantom+ MR	
	Pearson *r* value	*p*‐value	Pearson *r* value	*p*‐value
Area	0.39	0.02	**‐0.56**	**0.00**
Number of Active MLCs	0.34	0.04	**‐0.67**	**0.00**
Perimeter	0.35	0.04	**‐0.69**	**0.00**
Edge Metric	0.34	0.04	**‐0.68**	**0.00**
Perimeter Area Ratio	**‐0.56**	**0.00**	0.21	0.23
Average Leaf Pair Opening	0.39	0.02	‐0.24	0.15
Aperture Area Variability	0.17	0.33	0.48	0.00
Leaf Segment Variability	0.44	0.01	**‐0.57**	**0.00**
Complete Irradiation Area Outline	0.32	0.06	**‐0.69**	**0.00**
Modulation Complexity Score	0.38	0.02	0.24	0.15
Irregularity	0.08	0.63	**‐0.73**	**0.00**
Leaf Travel Index	‐0.20	0.23	**0.67**	**0.00**
Leaf Travel Index Modulation Complexity Score	0.35	0.04	0.27	0.11
Unique Opening Index	‐0.02	0.89	**‐0.64**	**0.00**
Cross Axis Score	‐0.29	0.09	0.29	0.09
Small Aperture Score (5 mm)	‐0.37	0.03	0.06	0.72
Small Aperture Score (10 mm)	‐0.35	0.04	0.00	0.99
Small Aperture Score (20 mm)	**‐0.53**	**0.00**	0.12	0.50
Mean Aperture Displacement	‐0.08	0.64	‐0.22	0.20
Closed Leaf Score	‐0.13	0.46	‐0.20	0.25
Number of Segments	‐0.02	0.89	‐0.18	0.29
Number of Beams	0.19	0.27	‐0.31	0.06
MU per Segment	‐0.04	0.83	0.35	0.03
Plan MU	‐0.22	0.20	‐0.14	0.41

**Fig. 3:**
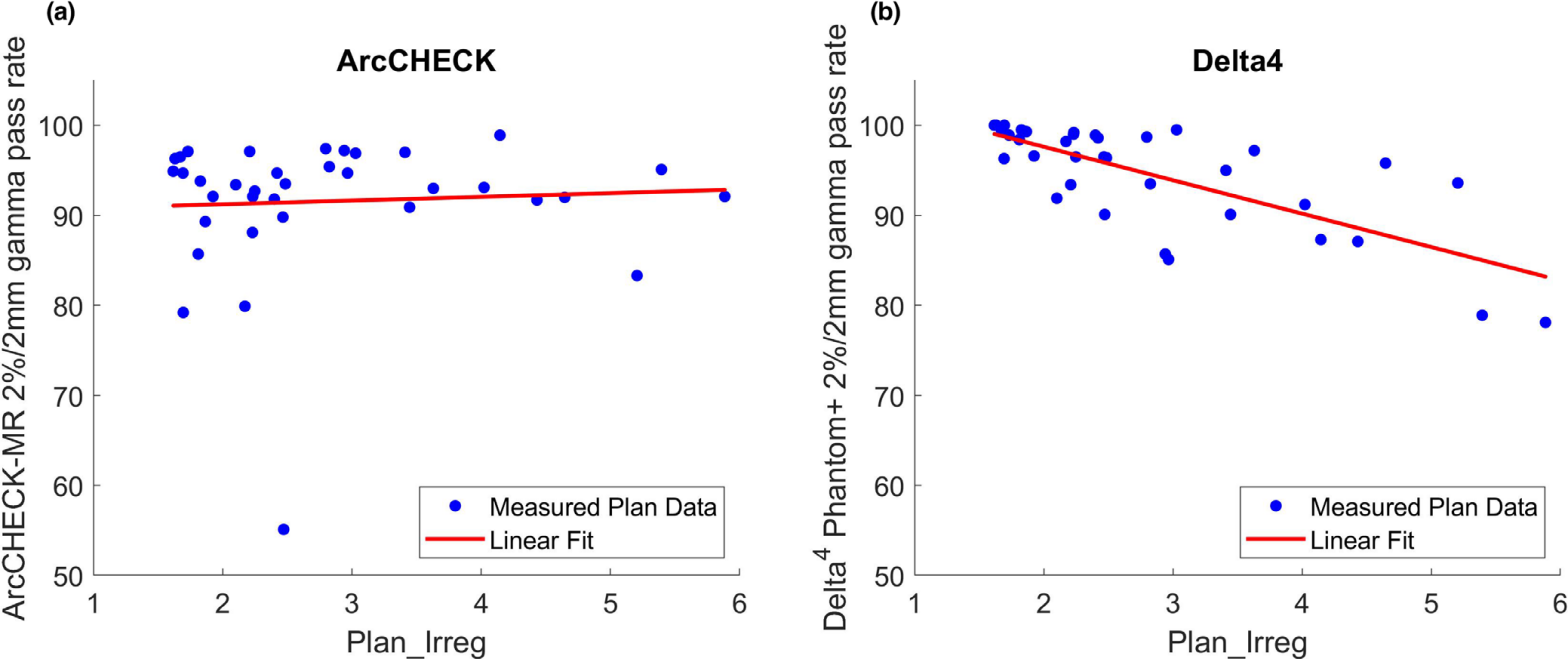
Comparison of 2%/2mm global gamma pass rate plotted as a function of the irregularity metric for both the (a) ArcCHECK‐MR and (b) Delta^4^ Phantom+ MR. Note that the irregularity metric is a unitless quantity.

The ArcCHECK‐MR 2%/2mm gamma pass rates generally showed weak correlation with most of the plan metrics investigated with only the small aperture score (20 mm) and perimeter to area ratio metrics appearing to show moderate negative correlation with the overall pass rate. The Delta^4^ Phantom+ MR 2%/2mm gamma pass rate, however, demonstrated stronger correlation with several plan metrics relating to field size like aperture area, total number of active MLCs, perimeter, complete irradiation area outline, and leaf travel, indicating that larger treatment fields were moderately negatively correlated with 2%/2mm global gamma pass rates. The irregularity metric originally developed by Du et al.^15^ was found to have the highest negative correlation with overall gamma pass rate for the Delta^4^ Phantom+ MR, however, it showed almost no correlation with the ArcCHECK‐MR results. Figure 3 compares the 2%/2mm global gamma pass rate across all 36 clinical plans for the Delta^4^ Phantom+ MR plotted as a function of the irregularity metric for both radiation detectors.

**Fig. 4:**
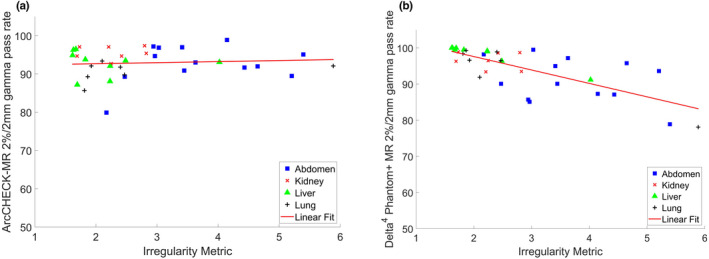
Comparison of 2%/2mm global gamma pass rate plotted as a function of the irregularity metric for both the (a) ArcCHECK‐MR and (b) Delta^4^ Phantom+ MR. Note that the irregularity metric is a unitless quantity. Data are further stratified based on the anatomical site of treatment.

Figure 4 shows the same plotted data as Fig. 3 but distinguishes each data point based on the anatomical site of treatment. By further classifying the data into these anatomical groups, it became possible to see which specific plans contributed the most to the correlation between a given metric and the 2%/2mm global gamma pass rates. For example, Fig. 4b shows that the liver and lung data largely drive the negative correlation seen between the Delta^4^ Phantom+ MR 2%/2mm global gamma pass rates and the irregularity metric. Conversely, the kidney plans measured using the Delta^4^ Phantom+ MR had almost no sensitivity to the irregularity metric (but also exhibited a noticeably narrower range of plan irregularity) and the abdomen data showed weak correlation with plan irregularity.

Table 6 classifies all 36 clinical plans based on the anatomical site of treatment and further evaluates each subgroup in the context of every complexity metric originally shown in Table 5. The 2%/2mm global gamma pass rates for the 13 abdominal plans were found to be significantly correlated with 12 out of 24 metrics for the ArcCHECK‐MR and none of the metrics were significantly correlated with the Delta^4^ Phantom+ MR abdomen data. For the seven kidney plans, only one metric (albeit different for each device) was found to be significantly correlated with the ArcCHECK‐MR and Delta^4^ Phantom+ MR 2%/2mm global gamma pass rates. For the liver data, 2 of 24 metrics were significantly correlated with the ArcCHECK‐MR gamma pass rates and 12 of 24 metrics were significantly correlated with the Delta^4^ Phantom+ MR gamma pass rates. Finally, the lung plan gamma pass rates showed no significant correlation with any plan metrics for the ArcCHECK‐MR but did show correlation with 9 of 24 metrics for the Delta^4^ Phantom+ MR.

**Table 6:** Summary of Pearson correlation coefficients with the 2%/2mm global gamma pass rate for both the ArcCHECK‐MR and the Delta^4^ Phantom+ MR with the data being further classified based on anatomical site of treatment. Correlation values with a magnitude larger than 0.5 and an associated p‐value of < 0.05 are highlighted in bold.

Metric	Pearson r correlation values							
	Abdomen		Kidney		Liver		Lung	
	ArcCHECK‐MR	Delta^4^ Phantom+ MR	ArcCHECK‐MR	Delta^4^ Phantom+ MR	ArcCHECK‐MR	Delta^4^ Phantom+ MR	ArcCHECK‐MR	Delta^4^ Phantom+ MR
Area	**0.60**	‐0.32	‐0.29	0.02	0.17	**‐0.96**	0.42	**‐0.96**
Number of Active MLCs	**0.62**	‐0.39	‐0.53	‐0.14	0.26	**‐0.88**	0.36	**‐0.97**
Perimeter	0.64	‐0.39	‐0.26	0.01	0.16	**‐0.97**	0.38	**‐0.96**
Edge Metric	**0.64**	‐0.38	‐0.01	0.12	0.11	**‐0.98**	0.39	**‐0.95**
Perimeter Area Ratio	**‐0.71**	0.24	0.23	0.06	‐0.53	0.62	‐0.51	0.47
Average Leaf Pair Opening	**0.66**	‐0.25	‐0.17	0.04	0.18	**‐0.94**	0.49	‐0.23
Aperture Area Variability	0.40	‐0.05	‐0.72	‐0.27	0.66	0.31	‐0.29	0.66
Leaf Segment Variability	0.75	‐0.42	‐0.72	0.05	0.33	**‐0.69**	0.37	**‐0.76**
Complete Irradiation Area Outline	0.60	‐0.40	‐0.12	0.29	0.11	**‐0.99**	0.34	**‐0.95**
Modulation Complexity Score	**0.64**	‐0.17	‐0.71	‐0.19	**0.73**	‐0.13	‐0.04	0.41
Irregularity	0.26	‐0.34	0.08	‐0.18	‐0.11	**‐0.97**	0.32	**‐0.94**
Leaf Travel Index	**‐0.60**	0.14	‐0.05	‐0.73	0.08	**0.97**	‐0.35	**0.91**
Leaf Travel Index Modulation Complexity Score	**0.62**	‐0.17	‐0.62	‐0.34	**0.74**	‐0.07	‐0.07	0.46
Unique Opening Index	‐0.02	‐0.18	0.17	‐0.37	‐0.31	**‐0.89**	0.29	**‐0.94**
Cross Axis Score	**‐0.61**	0.13	0.40	**0.79**	‐0.04	**0.68**	‐0.52	0.10
Small Aperture Score (5 mm)	**‐0.77**	0.24	0.37	‐0.56	‐0.19	0.21	0.30	0.16
Small Aperture Score (10 mm)	**‐0.61**	0.24	0.40	‐0.52	‐0.55	0.16	0.20	0.42
Small Aperture Score (20 mm)	**‐0.71**	0.24	0.25	‐0.21	‐0.64	0.32	‐0.42	0.44
Mean Aperture Displacement	‐0.23	0.06	‐0.13	‐0.36	0.11	‐0.20	0.20	‐0.54
Closed Leaf Score	‐0.52	0.17	**0.83**	0.02	‐0.60	‐0.07	0.28	‐0.18
Number of Segments	‐0.04	‐0.03	‐0.03	0.06	‐0.60	0.07	0.57	0.00
Number of Beams	0.51	‐0.39	0.46	0.28	‐0.24	**‐0.76**	‐0.27	0.04
MU per Segment	‐0.38	0.05	0.74	‐0.40	0.47	0.51	0.03	0.32
Plan MU	‐0.28	0.10	0.53	0.05	‐0.60	‐0.56	‐0.14	‐0.03

## 4 Discussion

The performance of the Delta^4^ Phantom+ MR was assessed through measurement of 36 clinical cases treated on a ViewRay MRIdian linac. The results were compared to measurements conducted using a Sun Nuclear ArcCHECK‐MR and found to be similar to one another. Both devices were found to be compatible with our institution’s 95% pass rate at 3%/3mm gamma criterion. More nuanced differences between the devices arose when considering the 2%/2mm global gamma pass rates. Three adaptive plans from this work (abdomen plan indices 11 and 12 and liver plan index 9) were specifically selected because it was known that the historical gamma pass rates on the ArcCHECK‐MR were abnormally low (i.e., <97% global gamma pass rate for 3%/3mm criteria). This type of comparison was only enabled by the fact that our institution has a longer history using the ArcCHECK‐MR phantom, so it was possible to pull out a few plans with less‐than‐ideal measurement results. In those three cases, the analogous Delta^4^ Phantom+ MR 3%/3mm global gamma pass rates were all higher than 99%.

For the 3%/3mm global gamma pass rates, the Delta^4^ Phantom+ MR measured higher pass rates than the ArcCHECK‐MR in 29 plans, lower pass rates in 6 plans, and an identical pass rate in 1 plan. Figure 2a illustrates that the distinction of having “higher” 3%/3mm global gamma pass rates is largely irrelevant since both devices measured results well above our institutional criteria and that the measured 3%/3mm pass rates were similar for both devices, except for the specific ArcCHECK‐MR low pass rate plans deliberately chosen from historical IMRT QA data. When considering the 2%/2mm global gamma pass rates, the Delta^4^ Phantom+ MR measured higher pass rates in 24 plans and lower pass rates in 12 plans than the ArcCHECK‐MR device. Here, more pronounced differences were observed between the detectors. Importantly, the authors strongly emphasize that favoring a detector based solely on which one measures a higher gamma pass rate is a fundamentally flawed perspective and is not a suitable “apples‐to‐apples” comparison. The validation performed in this work was based on clinical plans measured at different time points by different users. The variability in machine output, user setup, detector conditions, etc. all play a role in the final measured gamma pass rates but cannot be easily decoupled from the final results. Furthermore, the factors being considered when choosing an IMRT QA detector will differ for every institution including previous experience, budget, ease of use, as well as the sensitivities and specificities appropriate for the institution’s specific IMRT QA criteria. Therefore, the gamma pass rates reported in this work should be construed as a validation of the newer Delta^4^ Phantom+ MR detector and not as an absolute means of comparing it against the ArcCHECK‐MR.

A corollary to not solely relying on global gamma pass rates to assess an IMRT QA radiation detector, however, is the desire to understand why potential differences could arise from different detectors in ostensibly similar circumstances and what to make of them. Work is presently ongoing with ScandiDos to attempt to establish detector‐specific sensitivities to more clearly explain some of the results encountered but no obvious detector‐specific explanation has been determined through these efforts as to why gamma pass rates could potentially be higher than the ArcCHECK‐MR pass rates in some clinical situations, and lower in others. It is possible that some of the lower gamma pass rates seen from the larger IMRT fields measured on the Delta^4^ Phantom+ MR could be due to the lower resolution diodes (spaced 1 cm apart) necessarily being engaged in the large composite field size but those diodes are less equipped to handle steep dose gradients than the high resolution (spaced 0.5 cm apart) diodes in the central 6 cm of the detector. Previous work by de Vries et al.^11^ demonstrated up to an almost 3% under response of the Delta^4^ Phantom+ MR at very small static field sizes but showed that larger fields up to 22 cm 22 cm were still within 0.5% of reference conditions. The patching together of large fields in an IMRT delivery using numerous small segments was also considered as a possible source of lower gamma pass rates but a related quantity which was investigated, MU per segment, was only weakly correlated with the Delta^4^ Phantom+ MR 2%/2mm gamma pass rates. The ArcCHECK‐MR geometry, on the other hand, involves equally spaced diodes in a helical arrangement which could have a more uniform effect on the sampling rate in those same radiation fields, but the ArcCHECK‐MR also has fewer physical detectors in the vicinity of isocenter which could be disadvantageous in certain circumstances. With the clinical plans that were investigated, attributing differences in gamma pass rates solely to differences in detector construction and operation is a difficult task and was not performed in this work. An intuitive follow up study would be to investigate both detectors using even more clinical plans but also nonclinical fields which could be designed to evaluate each detector in nonideal fluence environments (e.g., beams entering perpendicularly to a specific diode board for the Delta^4^ Phantom+ MR).

The 2%/2mm gamma pass rates for both devices were also investigated as a function of 24 plan metrics. In the case of the ArcCHECK‐MR, weak correlation was found with most plan metrics investigated with only the small aperture score (20 mm) and perimeter area ratio metrics involving a statistically significant Pearson correlation coefficient magnitude of greater than 0.5. Assuming that measurement setup and machine behavior was relatively consistent between measurements, this suggests that the ArcCHECK‐MR response is mostly insensitive to the levels of fluence complexity investigated in this work. The Delta^4^ Phantom+ MR, however, did show moderate correlation with certain metrics including area, number of active MLCs, perimeter, edge metric, leaf segment variability, complete irradiation area outline, irregularity, leaf travel index, and unique opening index. Further investigation revealed that the liver and lung data measured using the Delta^4^ Phantom+ MR included several data points with lower gamma pass rates and higher levels of modulation, but no easily discernible plan feature or quality could help explain differences between the measured gamma pass rates measured using either the ArcCHECK‐MR or the Delta^4^ Phantom+ MR. Additionally, more pronounced differences between the detectors only arose when considering the more stringent and less clinically relevant 2%/2mm global gamma pass rates.

It should be emphasized that the complexity metrics calculated in this work are features of the plan fluence and completely independent of the radiation detector. The metrics are all averaged over the entirety of the composite IMRT plan and so much useful information is lost in the process of doing so. Frustratingly, the fundamental appeal of an ideal complexity metric is that it should be easy to calculate while still reliably predicting issues with a plan’s deliverability. To date, no such idealized metric has been identified or developed and the debate regarding the utility of patient‐specific QA measurement remains open‐ended. The 24 metrics explored in this work were investigated because they were easy to calculate and, in some way, quantitatively descriptive of the plan delivery, not because they were automatically assumed to be relevant IMRT QA measured using diode arrays. It is important to emphasize that causation is not inferred from these correlations and that more data are required prior to drawing any firm conclusions, however, it was still informative to include and investigate the plan metrics since they have been historically used to attempt to predict IMRT QA failure, and their relevance to ViewRay plans specifically has not been explored as much as other treatment delivery systems. Future work aims to make use of fluence complexity metrics in addition to the aperture complexity metrics used in this work since descriptors of the 3D fluence map could potentially relate measured gamma pass rates more directly to a detector’s specific geometry.

Further work characterizing the Delta^4^ Phantom+ MR detector and competing devices will help the community establish a body of data to ensure that the IMRT QA results being measured are consistent and accurate. Based on the results of this work, the Delta^4^ Phantom+ MR was found to be a reliable detector for clinical use with a 0.35T MR‐linac. Although not discussed in this work, using the Delta^4^ Phantom+ MR on a non‐MR linac, both in terms of calibration and measurements, was also straightforward and very similar to previous institutional experience with the standard Delta^4^ Phantom+. Therefore, institutions could potentially make use of a single Delta^4^ Phantom+ MR device for both MR‐ and conventional linear accelerators. Finally, modulation complexity metrics continue to be an actively researched topic with the goal of discovering a metric (or combination of metrics) truly capable of precluding the need for patient specific IMRT QA measurements in the first place, or at the very least, helping to identify potentially troublesome plans prior to a measurement being conducted at all. Their role in IMRT QA, however, remains questionable as no consistent metric or combination of metrics has been shown to universally predict IMRT QA failure, let alone more relevant quantities such as adverse patient outcomes.

## 5 Conclusions

Based on the results of this work, the Delta^4^ Phantom+ MR is found to be a reliable detector for clinical use with a 0.35T MR‐linac. The performance of the Delta^4^ Phantom+ MR is found to be comparable to a Sun Nuclear ArcCHECK‐MR device at the 3%/3mm global gamma level. Minor discrepancies between the two devices become more noticeable at the 2%/2mm global gamma level with the Delta^4^ Phantom+ MR gamma pass rates demonstrating moderate correlation with several plan complexity metrics. The ArcCHECK‐MR 2%/2mm global gamma results appear to be largely independent of the complexity of the radiation treatment plan.

## Conflict of Interest

The authors have no relevant conflicts of interest to disclose.
