# Erratum: “A Clinical Validation of the MR‐Compatible Delta^4^ QA System in a 0.35 tesla MR linear accelerator”

**DOI:** 10.1002/acm2.13878

**Published:** 2023-02-16

This erratum corrects the following:

Desai, V., Bayouth, J., Smilowitz, J. and Yadav, P. (2021), A clinical validation of the MR‐compatible Delta^4^ QA system in a 0.35 tesla MR linear accelerator. J Appl Clin Med Phys, 22: 82–91. https://doi.org/10.1002/acm2.13216 The authors report a change in the conflict of interest statement at the end of the article. The corrected conflict of interest statement is as follows:

## CONFLICT OF INTEREST

“John E. Bayouth reports ownership interest in a company that provides consulting services on image guided radiation therapy technology (MR Guidance, LLC,). He/(his employer) received travel reimbursement/speaking fees from ViewRay Inc. The remaining authors have no conflicts of interest to disclose. ”

